# Effect of cochlear implantation on cognitive decline and quality of life in younger and older adults with severe-to-profound hearing loss

**DOI:** 10.1007/s00405-022-07253-6

**Published:** 2022-01-19

**Authors:** Miryam Calvino, Isabel Sánchez-Cuadrado, Javier Gavilán, M. Auxiliadora Gutiérrez-Revilla, Rubén Polo, Luis Lassaletta

**Affiliations:** 1 Department of Otolaryngology, Hospital Universitario La Paz, IdiPAZ, Paseo de La Castellana, 261, 28046 Madrid, Spain; 2 Biomedical Research Networking Centre on Rare Diseases (CIBERER), Institute of Health Carlos III, U761 Madrid, Spain; 3 Department of Otolaryngology, Hospital Universitario Ramón y Cajal, Madrid, Spain

**Keywords:** Cochlear implant, Cognition, Age, Speech perception, Quality of life

## Abstract

**Purpose:**

(a) To measure the change in cognition, the improvement of speech perception, and the subjective benefit in people under and over 60 years following cochlear implantation. (b) To assess the relationship between cognition, demographic, audiometric, and subjective outcomes in both age groups.

**Methods:**

28 cochlear implant (CI) users were assigned to the < 60y group and 35 to the ≥ 60y group. Cognition was measured using the Repeatable Battery for the Assessment of Neuropsychological Status for Hearing impaired individuals (RBANS-H); subjective benefit was measured using the Nijmegen Cochlear Implant Questionnaire (NCIQ); the Glasgow Benefit Inventory (GBI); the Hearing Implant Sound Quality Index (HISQUI_19_); Speech, Spatial and Qualities of Hearing Scale (SSQ_12_); and the Hospital Anxiety and Depression Scale (HADS).

**Results:**

Prior to surgery: the RBANS-H total score positively correlated with the domains “Advanced sound”, “Self-esteem”, and “Social functioning” of NCIQ, and negatively with HADS scores. 12 months post-implantation: the RBANS-H total score increased in the < 60y (*p* = 0.038) and in the ≥ 60y group (*p* < 0.001); speech perception and subjective outcomes also improved; RBANS-H total score positively correlated with “Self-esteem” domain in NCIQ. Age and the RBANS-H total score correlated negatively in the ≥ 60y group (*p* = 0.026).

**Conclusions:**

After implantation, both age groups demonstrated improved cognition, speech perception and quality of life. Their depression scores decreased. Age was inversely associated with cognition.

## Introduction

Hearing loss is one of the leading contributors to years lived with a disability: over 5% of the world’s population–or 466 million people–have a disabling hearing loss. This number will increase as the population ages [1].

Besides, about 47 million people were living with dementia in 2015, and this number is expected to triple by 2050 [2]. On the other hand, people aged ≥ 60 years will grow to 2.1 million in 2050 [3]. Taking these facts into account, the challenge that is facing our society is not only to live longer but to live with fewer disabilities.

It was first stated in 1989 that hearing loss in elderly was strongly related to the risk of developing dementia [4]. Several studies have found a link between hearing loss and cognition in the elderly [5]. Nonetheless, no major conclusions have been drawn regarding this association further in the following years.

**Fig. 1:**
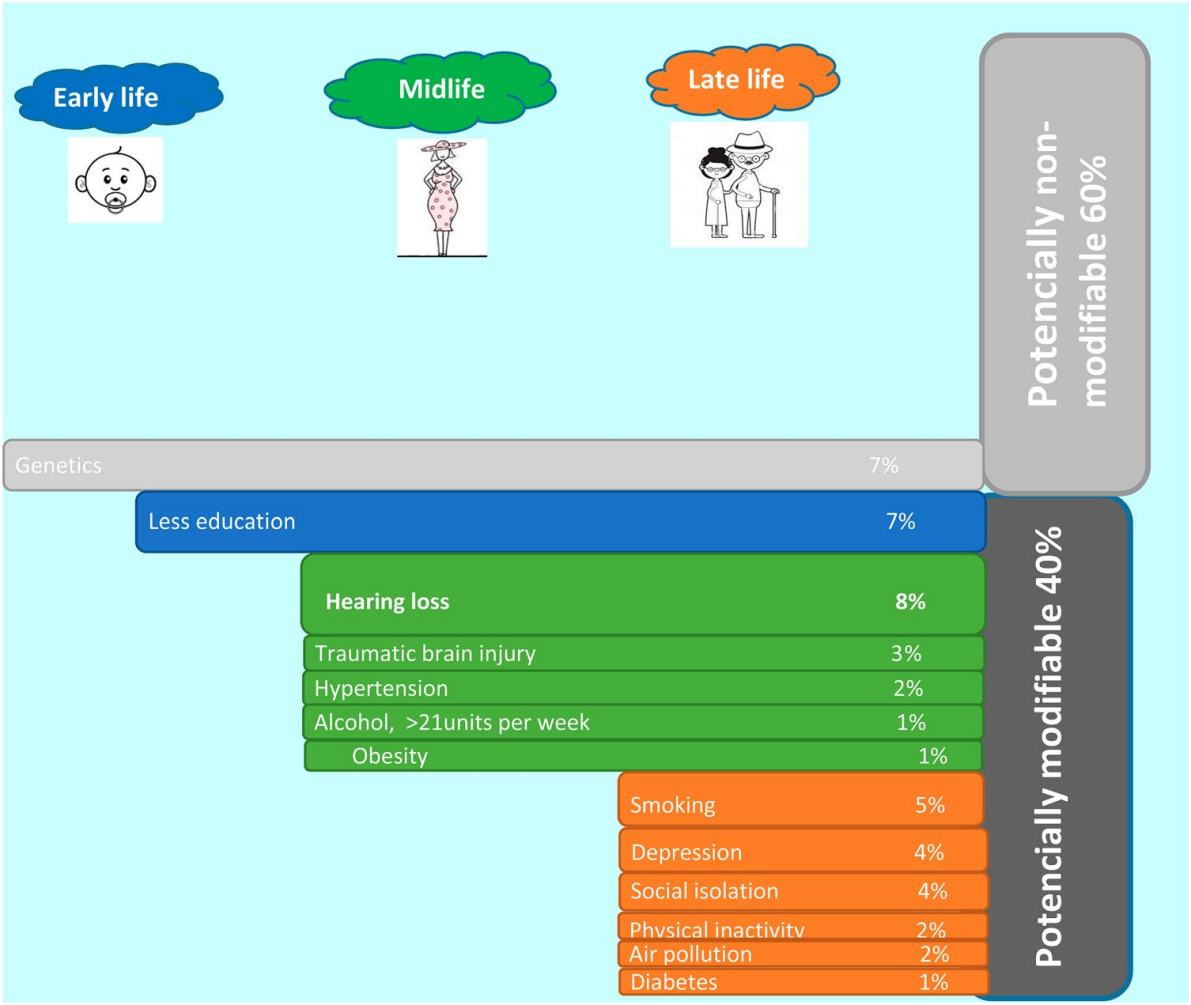
Non-modifiable and potentially modifiable risk factors of dementia across lifespan. Around 40% of cognition decline is may be explained by a mix of potentially modifiable risk factors: low educational level, hypertension, obesity, smoking, depression, physical inactivity, diabetes, low social contact, excessive alcohol consumption, air pollution, traumatic brain injury, and hearing loss. Conversely, genetics are believed to produce a 7% decrease in dementia incidence. Modified after [6, 45]

A recent report by the Lancet Commission on “dementia prevention, intervention and care” emphasizes strongly on prevention [6]. The authors of the report calculated that 40% of cases of mental decline could be prevented by modifying 12 risk factors as shown in Fig. 1*.* It was established that hearing loss accounted for 8% of dementia cases occurring during midlife.

There are various hypotheses linking deafness with cognitive decline and dementia. Despite being addressed individually, these hypotheses cannot be independent; it is assumed that several mechanisms, or all of them, may act in combination [7].

A. Cognitive load hypothesis: The high cognitive load of speech processing in people with hearing loss may accelerate neurodegeneration and brain atrophy. If cognitive resources are redirected to sensory processing, the resources available for cognitive processing are decreased, leading to cognitive decline.
B. Common cause hypothesis: Both hearing loss and cognitive decline may result from a common genetic or environmental cause.
C. Cascade hypothesis: Processing capacities may be lost when they are no longer used. Social isolation, depression, loneliness, reduced physical activity, and diminished quality of life may increase the vulnerability of people with a hearing loss and accelerate cognitive decline.
D. Overdiagnosis or harbinger hypothesis: Hearing loss might be misdiagnosed as cognitive decline because some cognitive tests rely on verbal abilities (i.e., instructions and questions are presented orally). On the other hand, hearing loss might be an early symptom of cognitive decline.

The last hypothesis leads to the assumption that correcting hearing loss with hearing aids or cochlear implants (CIs) could delay or even stop cognitive decline. Unfortunately, the results of the studies that measured the effect of hearing aids on cognition are inconclusive [8]. There are very few studies that looked at cognitive outcomes of CI users. Most of them only looked at the elderly and their results are inconclusive, too. In the review by Miller et al. [9] only three studies met the inclusion criteria (> 65 years, with a CI, post-implantation cognition as the primary outcome measure). In 2018, Claes et al. [10] reviewed the results of six studies: five of them reported improvements in cognition after implantation and one study did not observe any significant changes. More recently, Sarant et al. [11] concluded that cognitive function remained the same for 18 months in a subsample of 59 CI users with no cognitive impairment (age range: 61–89 years). In addition, it is imperative to emphasise the importance of a rehabilitation programme not only because it is crucial for the improvement of auditory performance with a CI, but because it may also play a role in the improvement in cognition [12].

The tests used to evaluate cognition are diverse and usually designed for people with normal hearing. The Repeatable Battery for the Assessment of Neuropsychological Status was first adapted for people with a hearing loss (RBANS-H) in 2016 [13]. In the RBANS-H, oral instructions are supported by written text in PowerPoint slides. Recently, Hillyer et al. [14] confirmed that visual presentation improves cognitive evaluation in people with hearing loss.

Based on these premises, the objectives of this study were:

- To measure the change in cognition after implantation in two groups of CI users (< 60 y and ≥ 60 y) using the RBANS-H.
- To measure speech perception and subjective benefit of CI use (including hearing performance, quality of life, mood disorders, and personality traits) in the same groups.
- To assess the relationship between cognition and demographic data, audiometric outcomes, and subjective benefit.

## Patients and methods

### Study design

A prospective study of consecutive CI candidates was conducted in the Departments of Otorhinolaryngology at La Paz University Hospital and Ramón y Cajal University Hospital, Madrid, Spain. The study procedures were approved by the Ethics Committees (PI-2755, 055-21).

Participants were enrolled in the study if they met the following inclusion criteria: (1) they were post-lingually deaf adults scheduled for their first cochlear implantation, (2) they had no neurological disease or cognitive impairment, and (3) they were willing to undergo an evaluation for approx. 1–1.5 h. Written informed consent was obtained from all of them. The procedure involved cognitive evaluation, audiological evaluation, and a series of questionnaires about the perceived benefit of CI use and quality of life. All evaluations except one were conducted twice: just before implantation and 12 months after first CI fitting. All participants were implanted with a MED-EL device (Concerto Mi1000 or Syncrony 1210) (MED-EL GmbH, Innsbruck, Austria).

Patients were divided into two age groups: < 60 years and ≥ 60 years. This cut-off was previously established in other studies that evaluated age-related hearing loss [15, 16].

### Procedures

#### Cognitive assessment

**Fig. 2:**
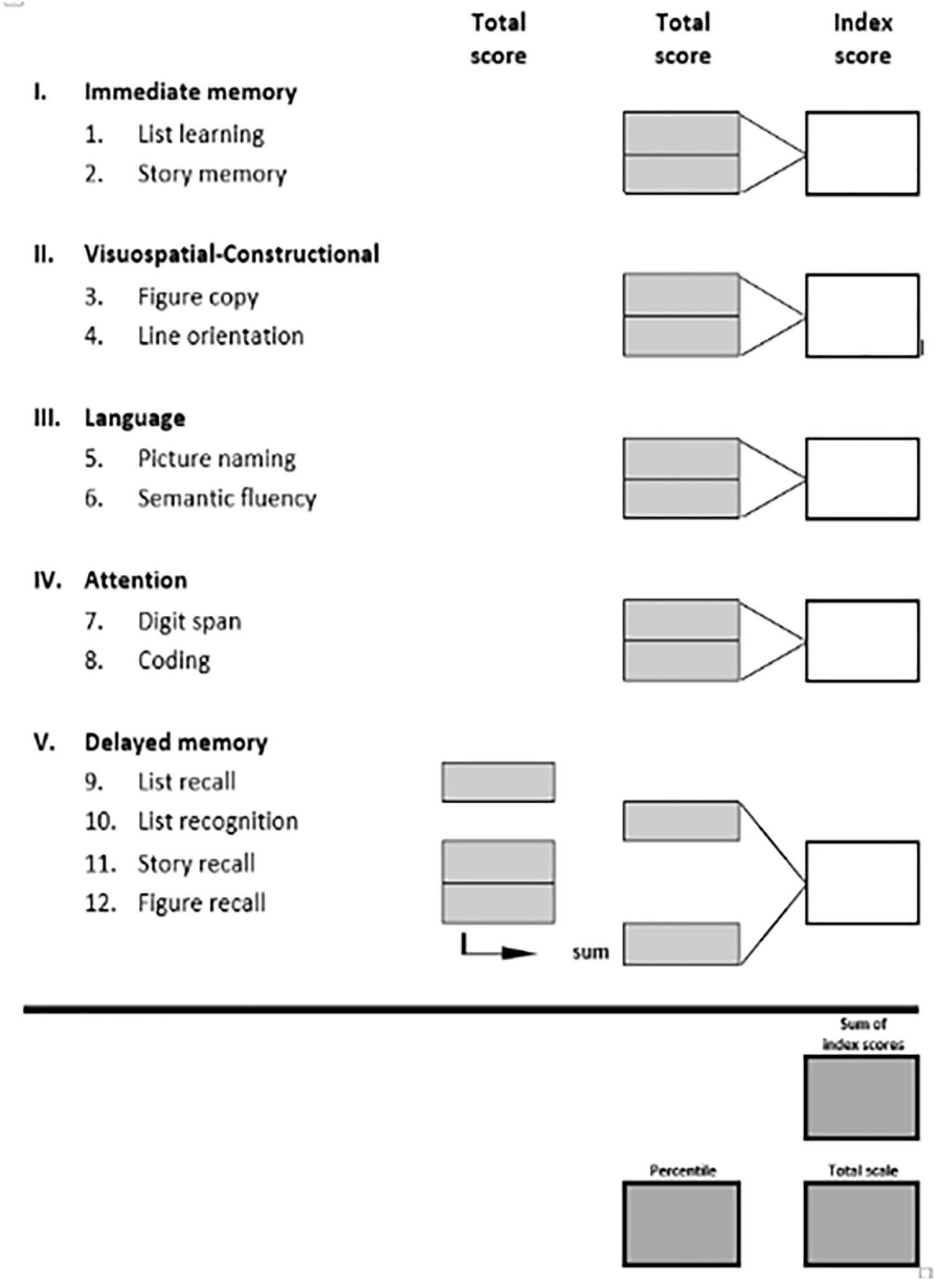
Score conversion sheet with the 5 domains (I-V) and the 12 subtests (1–12)

Cognitive abilities were measured using the Repeatable Battery for the Assessment of Neuropsychological Status for Hearing impaired individuals (RBANS-H) [13]. The measure was adapted for people with hearing loss by providing an audio-visual PowerPoint presentation with written instructions. It consists of 12 subtests that evaluate 5 cognitive domains: “Immediate memory,” “Visuospatial/constructional,” “Language,” “Attention,” and “Delayed memory” (Fig. 2). The hospital personnel involved in the study were trained to administer the RBANS-H (in Spanish) to reduce evaluator bias.

The total raw scores of the subtests were used to calculate the index score of each domain. These index scores were calculated using age-corrected tables with the following age categories: 12–13, 14–15, 16–19, 20–39, 40–49, 50–59, 60–69, 70–79, and 80–89 years. The final RBANS-H total score was calculated by the sum of these five index scores. This single total score was converted through a table to an age-corrected standard score with a mean of 100 and a standard deviation (SD) of 15 (see [13] for details). The age-corrected score is called “total scale” in the score conversion sheet (Fig. 2).

#### Audiological assessment

Audiological assessment was performed in a double-walled, soundproof booth using a two-channel Madsen Astera^2^ audiometer (Otometrics, Taastrup, Denmark). If a participant had better hearing in the non-implanted ear, this ear was masked during testing.

Pure-tone thresholds at 500, 1000, 2000, and 4000 Hz (PTA4) and the maximum Speech Discrimination Score (SDS) were measured before implantation under unaided conditions. After implantation, warble-tone thresholds in free field at 500, 1000, 2000, and 4000 Hz were measured using the CI. Speech perception was assessed with monosyllables, disyllables, and sentences in free field in quiet and noise. Participants were seated 1 m away from the loudspeakers at 0º azimuth. The tests were done without lip reading, at 65 dB SPL, and with a signal-to-noise ratio of 10 dB SPL speech-noise below the signal. The disyllable words are phonetically balanced words from the everyday vocabulary. The test was developed by Cárdenas de and Marrero [17]. The monosyllable test consists of lists of 20 words. The sentence evaluation consists of 100 sentences organised into 10 lists; this test is an adaptation of the "Every day sentences test" to Spanish [18]. Participants were asked to repeat the lists without any visual help. The results were presented as percentages.

#### Questionnaires

Subjective benefit of CI use was assessed using the Spanish versions of the following questionnaires: the Nijmegen Cochlear Implant Questionnaire (NCIQ); the Glasgow Benefit Inventory (GBI); the Hearing Implant Sound Quality Index (HISQUI_19_); Speech, Spatial and Qualities of Hearing Scale (SSQ_12_); and the Hospital Anxiety and Depression Scale (HADS). Except GBI that was only completed after implantation, all questionnaires were completed twice: before implantation and 1 year after first fitting.

##### NCIQ

The NCIQ is a validated closed-set questionnaire [19] comprised of 60 items. It was developed to evaluate the health effects of CI use. It has three general domains: physical, psychological, and social functioning. Each domain is divided into subdomains. The physical domain consists of basic sound perception, advanced sound perception, and speech production; the social domain consists of activity and social functioning; and the psychological functioning domain has only one subdomain—self-esteem. Each item was a statement with a 5-point response scale to indicate the degree to which the statement was true.

##### GBI

The GBI is a validated questionnaire developed to retrospectively assess the quality of life after otorhinolaryngologic interventions [20]. It consists of 18 questions and generates a scale from -100 (maximum detriment) through 0 (no change) to + 100 (maximum benefit). It assesses an individual’s perception of the overall success of CI use in terms of social and physical functioning (“Overall Benefit”, “General Health”, “Social Support”, and “Physical Health”).

##### HISQUI_19_

The HISQUI_19_ is a validated questionnaire [21] used to determine a CI user’s sound quality in daily life. It consists of 19 items with a 7-point Likert scale (1 – “never”, 7 – “always”). The scores of individual items are added together to produce a total score. A total score of 19‒29 indicates very poor sound quality; 30‒59‒poor sound quality; 60‒89‒moderate sound quality; 90‒109‒good sound quality, and 110‒133‒very good sound quality.

##### SSQ_12_

The SSQ_12_ is a validated [22] 12-item questionnaire that quantifies the severity of hearing disability. Individual items are answered on a 10-point Likert scale: the higher the score, the less disability is experienced. The total SSQ_12_ score (min: 0, max: 10) is the average of the individual item scores.

##### HADS

The HADS is a validated tool for measuring depression and anxiety [23]. It consists of 14 items: 7 items in the subscale “Depression” (e.g., “I can laugh and see the funny side of things”) and 7 items in the subscale “Anxiety” (e.g., “Worrying thoughts go through my mind”). Each item is answered on a four-point response scale from 0 to 3, so the total scores range from 0 to 21 in each subscale. 0–7 is a normal range, 8–10 indicates a borderline case, and a score of 11 or higher indicates a high probability of depression or anxiety (“caseness”) [23].

### Data analysis

To compare cognition (RBANS-H), audiometric data and self-reported outcomes (NICQ, GBI, HISQUI_19_, SSQ_12_, HADS) between the age groups (< 60y and ≥ 60y) at two time points (before and after implantation), the *t*-test (when the data were normally distributed) or the Mann–Whitney *U* test were used. To measure the difference within the groups, the *t*-test (when the data were normally distributed) or the Wilcoxon test were used. Normality was assessed by the Kolmogorov–Smirnov test and Q-Q plots.

Pearson’s correlation coefficient was independently calculated for the < 60y and the ≥ 60y group to evaluate the relationship between cognition (RBANS-H), age, audiometric data (PTA4 and speech perception test results), and the questionnaire scores (NICQ, GBI, HISQUI_19_, SSQ_12_, HADS). Analysis of variance (ANOVA) was used to measure the association between gender and cognition.

Missing data and the response option “Not applicable” were treated as missing values. A level of *p* ≤ 0.05 (2-tailed) was considered significant. Statistical analyses were done in the SPSS software package v24.0 (IBM Corp., Armonk, NY, USA).

Demographic characteristics and outcome measures are presented as absolute values, percentages and, where appropriate, the mean and ± SD are provided.

## Results

### Participants

63 participants were enrolled in the study: < 60 years (*n* = 28, mean age = 48.7 ± 8.3 years) and ≥ 60 years (*n* = 35, mean age = 70.5 ± 6.2 years). The demographic data of both groups are presented in Table 1.

**Table 1:** Demographic and pre-implantation audiometric data

*n*	< 60 years old	≥ 60 years old	*p*-value
	28	35	
Age (years) (mean ± SD) (range)	48.7 ± 8.3 (23–58)	70.5 ± 6.2 (60–82)	< 0.001
Gender (*n*) (%)			
Male	12 (43%)	20 (57%)	
Female	16 (57%)	15 (43%)	
HL aetiology (*n*) (%)			
Unknown	13 (46%)	16 (46%)	
Infection	2 (7%)	2 (6%)	
Congenital	2 (7%)	0 (0%)	
Ototoxicity	3 (11%)	1 (3%)	
Otosclerosis	4 (14%)	8 (23%)	
Trauma	3 (11%)	0 (0%)	
Meniere	1 (4%)	2 (6%)	
Meningitis	0 (0%)	2 (6%)	
Cholesteatoma	0 (0%)	4 (11%)	
HL duration (years) (mean ± SD)	22.7 ± 15.5	24.6 ± 15.2	0.951
Use of HA in the contralateral ear (n)	14 (50%)	20 (57%)	0.495
PTA4 (dB) in ear to be implanted*	134.9 ± 63.9	108.5 ± 20.6	0.527
Maximum SDS (%) in ear to be implanted	8.0 ± 14.2	17.9 ± 27.6	0.362
Use of CI at evaluation time (months) (mean ± SD)	12.2 ± 3.1	12.2 ± 1.0	0.985

*HA* hearing aid, *HL* hearing loss, *SD *standard deviation, *PTA4 *mean pure-tone audiometry values at 500 Hz, 1000 Hz, 2000 Hz, and 4000 Hz, *SDS *speech discrimination score. *When no response was detected, a value of 140 dB was used

### Cognitive status

**Fig. 3:**
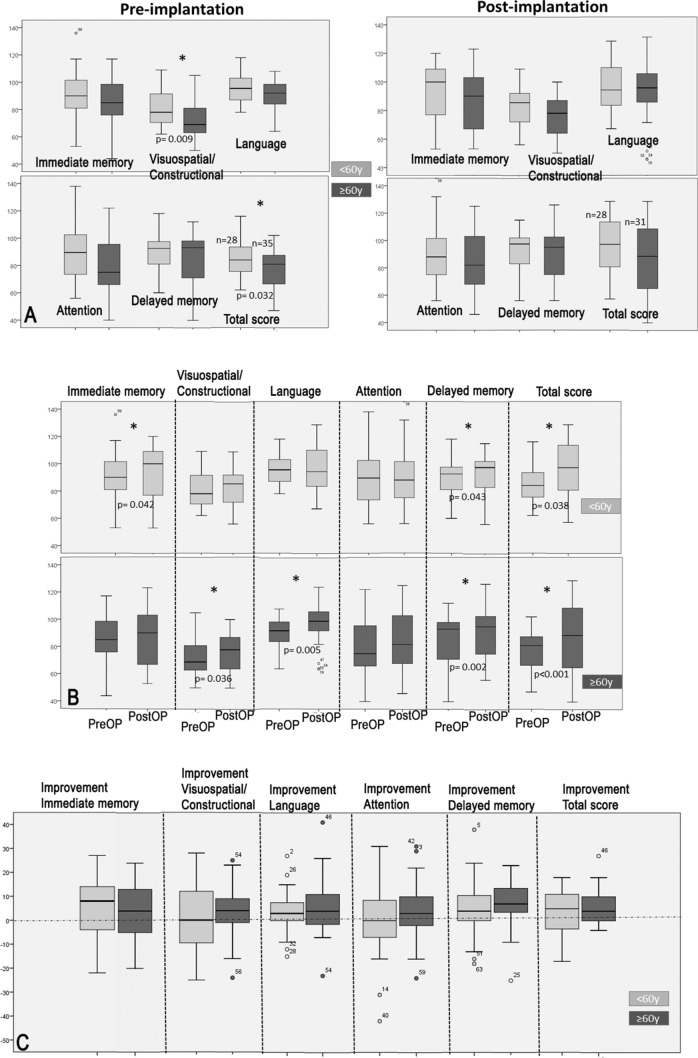
Cognitive abilities represented by the total Repeatable Battery for the Assessment of Neuropsychological Status for Hearing impaired individuals (RBANS-H) score and domain scores for the < 60y group (light grey) and the ≥ 60y group (dark grey). Higher scores indicate better cognition. The boxplots represent the minimum, 1st quartile, median, 3rd quartile, and maximum of the RBANS-H total and domain score before implantation (PreOP) and 12 months after implantation (PostOP). **A** Between-group comparison of the RBANS-H scores pre- and post-operatively. **B** Within-group comparison of the PreOP and PostOP RBANS-H scores. **C** Between-group comparison of the changes in the total and domain scores after implantation. *p < 0.05

Pre-implantation, the total RBANS-H score and the “Visuospatial/constructional” domain score were significantly higher in the < 60y group than in the ≥ 60y group (*p* = 0.032 and 0.009, respectively). No difference between the groups was found in the total RBANS-H or domain scores post-implantation (*t*-test) (Fig. 3A). Post-implantation, the RBANS-H total score improved significantly in both age groups (Fig. 3B). In the < 60y group, the “Immediate memory” and “Delayed memory” scores improved significantly (*p* = 0.042 and 0.043, respectively). In the ≥ 60y group, the Wilcoxon signed rank test revealed a significant increase in the “Visuospatial/constructional” (*p* = 0.036), “Language” (p = 0.005), and “Delayed memory” scores (*p* = 0.002).

#### Improvement of RBANS-H scores after cochlear implantation

The change in the RBANS-H scores after cochlear implantation was compared in both age groups (Fig. 3C). A positive value indicates an improvement in cognitive abilities, zero indicates no change, and a negative value indicates a decrease in cognitive abilities. Cognition scores improved in 64% in the < 60y group and in 71% in the ≥ 60y group. No change was observed in 16% in the ≥ 60y group. The total score increased on average by 3.9 ± 9.3 points in the < 60y group and by 5.8 ± 7.4 points in the ≥ 60y group. The difference in cognition improvement between the two groups was not statistically significant.

### Audiological assessment

The unaided PTA4 and SDS scores before implantation were similar in both age groups, *p* > 0.05 (Table 1). After implantation, all participants used their audio processors daily. Both groups had similar outcomes in all audiometric tests except the monosyllable test in quiet, in which the ≥ 60y group performed better than the < 60y group (*p* = 0.012) (Table 2).

**Table 2:** Post-implantation audiometric outcomes (% correct) in free field with the cochlear implant

	< 60y	≥ 60y	*p*-value
PTA4 (dB) (mean ± SD)	38.5 ± 12.6	34.8 ± 4.9	0.221
Monosyllabic words (%) (mean ± SD)			
Quiet	74.6 ± 18.2	60.0 ± 16.7	0.012*
Noise	69.6 ± 9.2	53.8 ± 22.6	0.089
Disyllabic words (%) (mean ± SD)			
Quiet	69.7 ± 19.7	66.8 ± 21.1	0.632
Noise	61.2 ± 17.6	52.2 ± 23.4	0.288
Sentences (%) (mean ± SD)			
Quiet	91.6 ± 17.3	90.0 ± 12.2	0.054
Noise	87.4 ± 21.1	82.1 ± 21.8	0.342

*Statistically significant, *p* < 0.05

### Subjective questionnaires

#### NCIQ

**Fig. 4:**
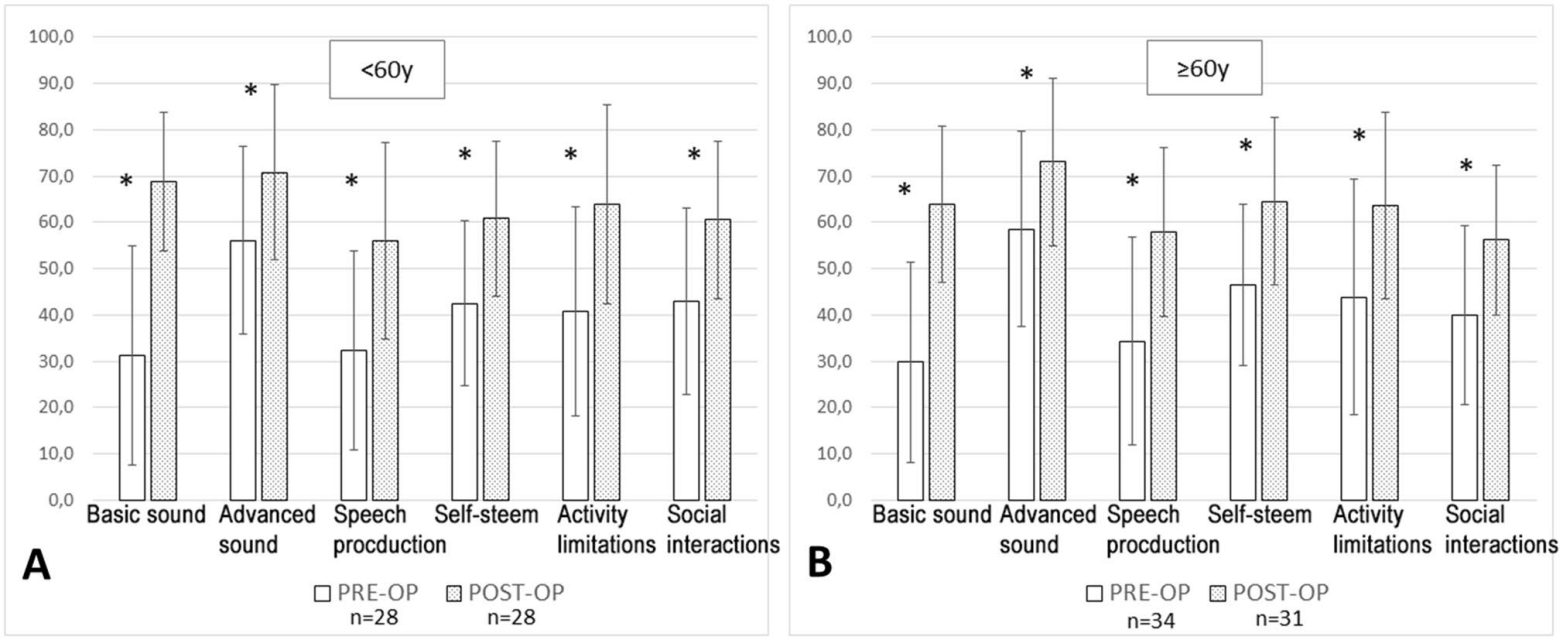
Preoperative (white) and postoperative (grey) Nijmegen Cochlear Implant Questionnaire (NCIQ) results in the < 60y group (**A**) and in the ≥ 60y group (**B**). *Statistically significant, *p* < 0.05

In both age groups, all NCIQ subdomain scores increased significantly after 12 months of CI use (Fig. 4A and B). There was no significant difference between the age groups (*t*-test).

#### GBI

On average, the overall GBI score and the general subscale score were highly positive (above 30) in both age groups (Table 3). In the two age groups combined, 93% of participants reported a positive overall change after cochlear implantation. The overall GBI scores and the subscale scores were similar in both groups.

**Table 3:** Mean Glasgow benefit inventory (GBI) scores after implantation

GBI score		Mean ± SD	N of participants (%)		
			Positive change	Negative change	No change
Overall	< 60y	30.4 ± 26.1	26 (93%)	2 (7%)	0 (0%)
	≥ 60y	36.9 ± 22.0	29 (93%)	2 (7%)	0 (0%)
Social subscale	< 60y	23.8 ± 24.2	15 (54%)	0 (0%)	11 (46%)
	≥ 60y	22.6 ± 25.7	18 (58%)	0 (0%)	13(42%)
Physical health subscale	< 60y	3.6 ± 17.2	7 (25%)	3 (11%)	18 (64%)
	≥ 60y	8.6 ± 26.1	7 (22%)	3 (10%)	21 (68%)
General subscale	< 60y	38.7 ± 35.7	26 (93%)	2 (7%)	0 (0%)
	≥ 60y	47.6 ± 30.2	29 (93%)	2 (7%)	0 (0%)

The questionnaire was completed by 100% of the < 60y group (*n* = 28) and by 89% (*n* = 31) of the ≥ 60y group

#### HISQUI_19_

**Fig. 5:**
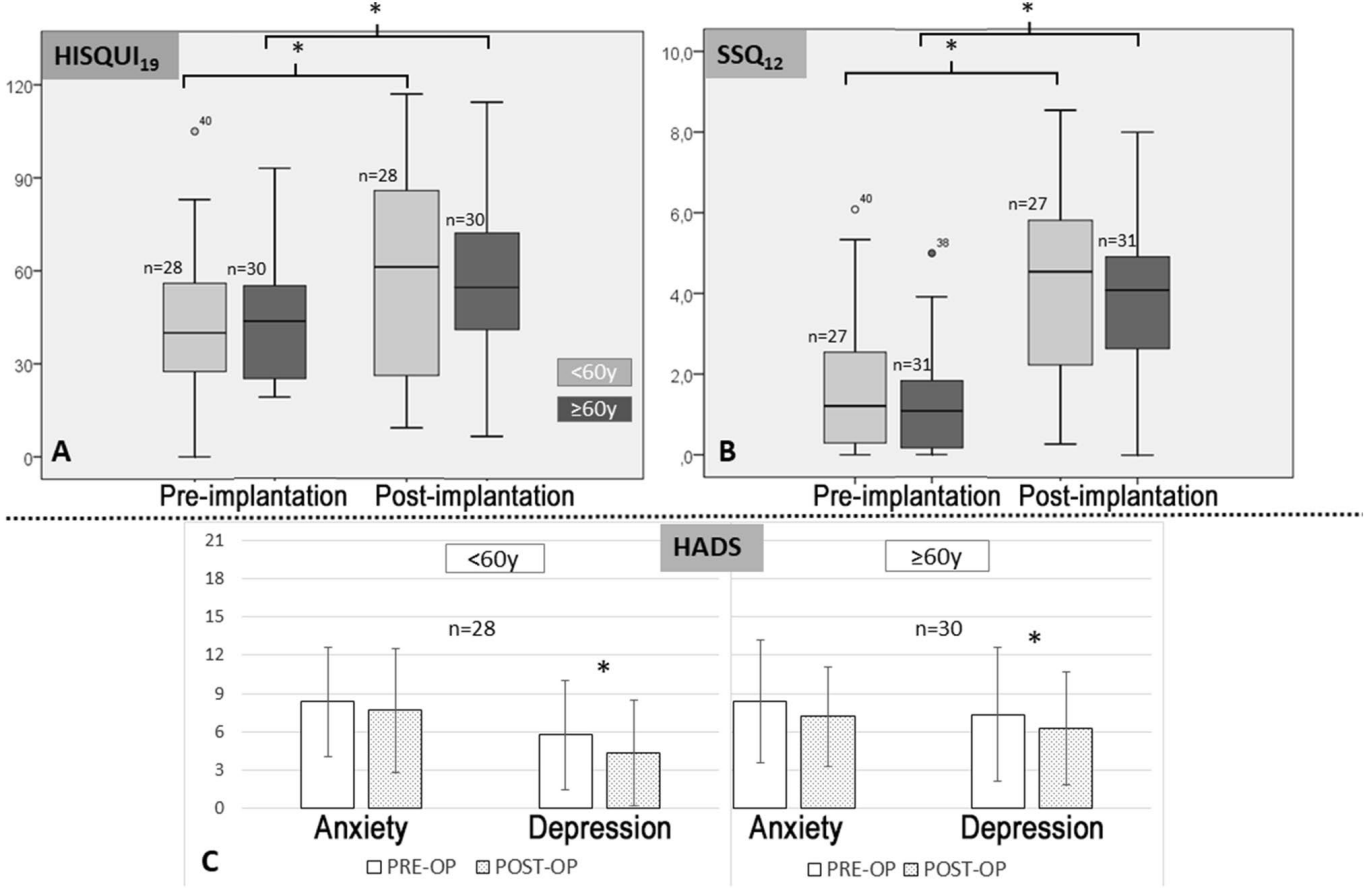
Pre- and post-implantation total scores. **A** Hearing Implant Sound Quality Index (HISQUI_19_). **B** Speech, Spatial and Qualities of Hearing Scale (SSQ_12_). **C** Hospital Anxiety and Depression Scale (HADS). *Statistically significant, *p* < 0.05

Post-implantation, both groups reported an improvement from “poor” sound quality to “moderate”. The scores in the < 60y group increased from 42.7 ± 22.1 to 74.8 ± 25.0, and from 44.5 ± 19.8 to 72.8 ± 18.8 in the ≥ 60y group (Fig. 5A). An improvement in sound quality was reported by 89% of participants in the < 60y group and by 93% in the ≥ 60y group. The results were similar in both groups.

#### SSQ_12_

The pre-implantation SSQ_12_ scores revealed a high level of perceived hearing disability in both groups (< 60y: 1.6 ± 1.7; ≥ 60y: 1.3 ± 1.3) (Fig. 5B). After 12 months of CI use, the mean scores in both groups increased significantly, indicating a moderate level of hearing disability (< 60y: 4.2 ± 2.3; ≥ 60y: 3.9 ± 1.9). Interestingly, the SSQ_12_ score improvement after cochlear implantation was the same in both age groups (2.5 ± 2.3). An improvement in perceived hearing disability was reported by 89% of participants in the < 60y group and by 87% in the ≥ 60y group.

#### HADS

Before implantation, the mean anxiety scores indicated a “borderline abnormal” anxiety level in both groups (< 60y: 8.4 ± 4.3; ≥ 60y: 8.3 ± 4.8) (Fig. 5C). After implantation, the anxiety level decreased to “normal” (< 8) in both groups (< 60y: 7.7 ± 4.8; ≥ 60y: 7.12 ± 3.9). A decrease in the anxiety scores was reported by 57% of participants in the < 60y group and by 67% in the ≥ 60y group.

After 1 year of CI use, the mean depression scores decreased significantly in both age groups: from 5.8 ± 4.3 to 4.4 ± 4.2 in the < 60y group and from 7.3 ± 5.3 to 6.3 ± 4.4 in the ≥ 60y group (Fig. 5C). A decrease in the depression scores was reported by 82% of participants in the < 60 group and by 50% in the ≥ 60y group.

### Relationship between cognition and demographic, audiometric, and subjective benefit outcomes

#### Relationship between RBANS-H scores and demographic data

##### Gender

Pre-implantation, men in the < 60y group had higher scores than women in the “Attention” domain (100.8 ± 14.1 vs 83.2 ± 22.4, *p* = 0.024). Men in the ≥ 60y group had higher scores than women in the “Visuospatial/constructional” domain (77.5 ± 14.2 vs 67.1 ± 11.0, *p* = 0.020) and in the “Attention” domain (89.9 ± 23.3 vs 73.2 ± 21.4, *p* = 0.034).

##### Age

Pre-implantation, the age in the ≥ 60y group had a significant negative correlation with the “Delayed Memory” domain (*p* = 0.023). Post-implantation, the age in the same group negatively correlated with the total RBANS-H score (*p* = 0.023), the “Visuospatial/constructional” (*p* = 0.010), “Attention” (*p* = 0.041), and “Delayed Memory” (*p* = 0.019) domain scores. In this group, age had a significant negative correlation with the improvement in the total RBANS-H score (*p* = 0.026).

#### Relationship between RBANS-H scores and audiometric outcomes

Some positive correlations between cognition and the audiometric outcomes were found in the ≥ 60y group. Pre-implantation, the “Immediate memory” domain score correlated with the outcomes of the sentence test in noise (*p* = 0.032). Post-implantation, the “Visuospatial/constructional” domain score correlated with the monosyllable test in silence (*p* = 0.029) and noise (*p* = 0.033), the disyllable test in silence (*p* = 0.007), and the sentence test in noise (*p* = 0.021).

#### Relationship between RBANS-H scores and subjective benefit questionnaires

Some positive correlations were found between the subjective benefit scores and the total RBANS-H score only in the ≥ 60y group (Table 4). Pre-implantation, the total RBANS-H score had a positive correlation with the “Advanced sound”, “Self-esteem”, and “Social functioning” domains in the NCIQ. Cognition also had a negative correlation with all subscales of the HADS, suggesting that better cognition is associated with lower anxiety and depression. Post-implantation, cognition had a positive correlation with the “Self-esteem” domain in the NCIQ.

**Table 4:** Correlations between the RBANS-H total score and the subjective benefit questionnaires in the ≥ 60y group

	NCIQ						HADS			
	Advanced sound		Self-steem		Social		Anxiety		Depression	
	PreOP	PostOP	PreOP	PostOP	PreOP	PostOP	PreOP	PostOP	PreOP	PostOP
PreOP RBANS-H total score	**0.431** **0.011**	NS	**0.480** **0.004**	NS	**0.408** **0.017**	NS	**-0.498** **0.003**	NS	**-0.548** **0.001**	NS
PostOP RBANS-H total score	NS	NS	NS	**0.397** **0.030**	NS	NS	NS	NS	NS	NS

First line—Pearson`s coefficient, second line—significance level (*p* < 0.05). *NS *not significant, *PreOP *pre-operative, *PostOP *post-operative

## Discussion

In this study, we demonstrated a significant improvement in cognition after 12 months of CI use in people with severe-to-profound hearing loss under and over 60 years old. To measure cognition, we used a version of the RBANS questionnaire specially adapted for people with hearing loss. Before implantation, the < 60y group had higher total RBANS-H scores than the ≥ 60y group, but after implantation both groups had similar scores (with the improvement in cognition being higher in the ≥ 60y group).

We also demonstrated an improvement in speech perception, besides perceived sound quality, and quality of life using the NCIQ, GBI, HISQUI_19_, SSQ_12_, and HADS questionnaires. These subjective outcomes along with gender, and age were associated with the RBANS-H cognition scores.

### Cognitive evaluation in people with hearing loss

In recent years, there has been a growing interest in the evaluation of cognitive abilities. The most widely used tools to measure cognition in research and clinical trials are the Mini-Mental State Examination (MMSE), the Montreal Cognitive Assessment (MoCA), the Mini-Cog test, the Addenbrooke's Cognitive Examination-Revised (ACE-R) [24], and the dementia-detection test (DemTect) [25]. These tests rely on oral instructions, i.e., they implicitly assume that the test subjects have normal hearing. Recent studies [26] have concluded that cognitive decline may be misdiagnosed in people with hearing loss. Besides, an increase in cognition scores after cochlear implantation may simply indicate improved hearing and not improved cognitive abilities as such. Hearing words with difficulty may decrease the cognitive resources needed to remember them correctly [27]. These facts support the last hypothesis (overdiagnosis or harbinger hypothesis) discussed in the introduction section. Therefore, CI candidates need an adequate cognitive evaluation tool. The RBANS-H test used in this study provides a mix of written and oral instructions in a PowerPoint presentation, so it avoids underestimating cognitive performance in people with hearing loss.

### Effect of cochlear implantation on cognitive outcomes

The first objective of our study was to measure the changes in cognitive performance 1 year after CI surgery in two age groups of people with severe-to-profound hearing loss. Some authors have previously used the RBANS-H to measure cognitive performance in people with hearing loss: age ≥ 55y [28], age range 58-94y [13], age range 55-85y [29], and mean age 72y [30]; but ours is the first study that looked at two different age groups, including CI users under 55 years.

In the present study, both “Memory” domains (“Immediate memory” and “Delayed memory”) significantly improved in the < 60y group, while the “Delayed memory”, “Visuospatial/ constructional” and “Language” domains improved in the ≥ 60y group. Our results are similar to those reported by Claes et al. [10], and Mertens et al. [28]. In both studies, a significant change in overall cognition, “Immediate memory”, “Attention”, and “Delayed memory” was demonstrated after 12 months of CI use. The improvement of the total RBANS-H scores in our study was slightly lower than in those mentioned above, suggesting that additional factors may be associated with cognitive decline dementia apart from hearing loss. Zhan et al. [31] reported an improvement in several cognitive tasks (working memory, concentration, and information processing speed) 6 months after implantation using a battery of visual tests. That study did not include different age groups, though (age range: 49–82 years). Similarly, Vasil et al. [32] administered the MoCA using audiovisual instructions and demonstrated an increase of the scores in CI users between 55 and 85 years old. In the present study, only a few participants showed a decrease in their RBANS-H scores after implantation, the percentage being higher in the < 60y group (35% vs 11%). No improvement in cognition after cochlear implantation was previously reported by Ambert-Dahan et al. [33], who evaluated cognitive performance with an adapted visual presentation of the MoCA test.

### Effect of cochlear implantation on audiological and subjective outcomes

The second objective of our study was to measure the changes in speech perception and assess the subjective benefit of CI use in two age groups 1 year post-implantation. We demonstrated that cochlear implantation led to better speech perception and increased quality of life as measured by the NCIQ, GBI, HISQUI_19_, SSQ_12_, and HADS questionnaires.

#### Effect of cochlear implantation on speech perception

According to previous research, cochlear implantation leads to better speech discrimination in a wide range of ages. Unlike Völter et al. [34], who found no difference between the two age groups (50–64y and > 65y), we found that the ≥ 60y group showed better results than the < 60y group in the monosyllable test in silence. This finding is difficult to compare with other studies because they used different speech tests. In a recent meta-analysis of 13 studies with 1095 participants, age at implantation was not associated with CI speech perception outcomes [35]. However, other studies have suggested age as a predictive factor for post-implantation performance [36].

#### Effect of cochlear implantation on quality of life

Although hearing restoration is the main aim of cochlear implantation, CI use has a wider impact on the user’s quality of life. In this study, higher scores were found in the NCIQ, GBI, HISQUI_19_, and SSQ_12_ questionnaires in both age groups after implantation. This perceived increase in quality of life has been demonstrated by our team [37], and by others [34] in people of different ages.

More specifically, Völter et al. [34] found that quality of life measured using the NCIQ was equal between the two groups (50–64y and > 65y) except for the basic sound perception domain, in which the older group had better pre-implantation results. But those authors used only the NCIQ to evaluate quality of life, whereas we assessed several subjective outcomes, thus covering various day-to-day tasks of CI users.

#### Effect of cochlear implantation on depression and anxiety (HADS)

People with hearing loss are more likely to have multiple psychological challenges such as anxiety and social isolation. In addition, hearing loss increases the risk of depression [38]. The present study showed a post-implantation decrease in anxiety and depression in more than half of the participants in both age groups. The mean anxiety values went from “borderline” to “normal” and the depression scores decreased significantly. Interestingly, the ≥ 60y group showed higher depression scores than the < 60y group after implantation, suggesting that aging may play a role in depression. A recent systematic review [38] mentioned two studies (mean ages: 58.6 and 51.7 years) in which cochlear implantation reduced the symptoms of depression. Bergman et al. [39] found that the level of depression decreased 1 year after implantation in a group of CI users with a median age of 72 years. Conversely, Mertens et al. [28] found no effect of CI use on the levels of anxiety and depression in 24 CI users (mean age: 72 years).

### Relationship between cognition and demographic, audiological, and subjective outcomes

The third objective of this study was to analyse the relationship between cognition and the demographic variables (age, gender), the audiological outcomes, and the subjective benefit of CI use.

#### Demographic variables

##### Gender

In this study, men had better cognition scores than women in some RBANS-H domains before implantation.

Gender has been associated with cognitive abilities. There is some evidence that women might be more likely than men to develop dementia [40]. Sarant et al. [11] found that executive function (evaluated with the Cogstate Brief Battery and the Cogstate Groton Maze Learning Test) improved significantly in males 18 months after cochlear implantation. Völter et al. [41] found that women outperformed men in a subtest on verbal fluency.

##### Age

Cognitive declines and the associated neuropathological changes are clearly age-related [6]. In our study, age had a significant correlation with cognition in the ≥ 60y group, i.e., the older a CI user was, the poorer their cognitive outcomes were. These findings are in agreement with Völter et al. [34], who also found that age had a much stronger association with cognition in the older group (≥ 65 years) and only a weak association in the younger group (50–64 years). Other age-related factors such as chronic conditions and unhealthy habits may also contribute to cognitive decline [6].

#### Speech perception

We found an association between audiological outcomes and two cognitive domains in the ≥ 60y group. In this age group, better results in the sentence test in noise corresponded to better scores in the “Immediate memory” domain pre-implantation. Post-implantation, several speech tests correlated with the “Visuospatial/constructional” domain scores.

Some authors have tried to determine whether better cognitive abilities could predict better post-implantation hearing outcomes, since cognitive skills are thought to contribute to speech performance. This is even more important in challenging hearing conditions like listening with a CI, as there are less spectrum-temporal issues. However, to date the results have been inconclusive [31, 32, 42].

Recently, Vasil et al. [32] reported a correlation between the MoCA performance and speech perception in 55- to 85-year-old CI users. Zhan et al. [31], in a study with 19 CI candidates (mean age: 67.8 years) found that neurocognitive abilities such as working memory and inhibition significantly correlated with the aided speech perception results. Conversely, Völter et al. [42] found no correlation between pre-implantation cognition and post-implantation speech perception outcomes in quiet or noise (mean age: 65.8 years).

#### Subjective benefit of CI use (questionnaires)

We found several interesting interactions between cognition and the subjective benefit of CI use, mainly in the ≥ 60y group.

##### General quality of life

In the ≥ 60y group, the total RBANS-H score correlated with the “Advanced sound”, “Self-esteem”, and “Social” NCIQ domains pre-implantation, and with the “Self-esteem” domain post-implantation. In the study by Völter et al. [34], advanced sound perception was associated with the working memory in the younger group (50–64 years) and the improvement in working memory was associated with the post-implantation improvement of social relationships in the total sample. Self-esteem is considered an important factor in mental disorders (especially in people over 60 years old), so better self-esteem could probably be associated with better results in cognition tests, too.

##### Mood disorders

Social isolation may lead to anxiety and depression as well as to a decrease in cognitive abilities in CI candidates, and mental health problems could be the link between hearing loss and cognition. Therefore, depression might add cognitive problems in people with hearing loss [43]. This could explain why in our study higher scores in the anxiety, and depression subscales were associated with poorer cognition scores pre-implantation. Völter et al. [34] found that the depressive symptoms were not associated with cognition in the ≥ 65y group pre-implantation. We found no association between the mental health problems and cognitive decline after implantation. Mertens et al. [28] corroborated this finding.

### Limitations

A potential limitation of this study might be the learning effect on the RBANS-H scores, because the same version was used pre- and post-implantation. Individuals might have improved their cognition scores because they had already done the same exercises before. This limitation may be addressed using two different RBANS-H versions, although Claes et al. [10] stated that this would not be enough to completely eradicate the learning effect.

Despite that all patients had bilateral hearing impairment and similar duration of deafness, the precise benefit obtained from the use of one or two hearing aids prior to cochlear implantation could have been somehow different between both age groups. Although unlikely, this difference could have impacted the different baseline cognitive status.

Due to the nature of cognitive decline, it should be monitored over a long period of time. So, a longer follow-up interval after implantation would potentially be more informative, because the effects of CI use are not always observed in studies with short follow-up periods.

Future studies would benefit from taking other modifiable risk factors of cognitive decline defined by Livingston et al. [6] (such as diabetes, alcohol, obesity, smoking, or hypertension) into account.

### Recommendations

Hearing loss in the elderly is underdiagnosed and undertreated: almost 2/3 of people with hearing loss between 48 and 92 years old do not use hearing aids [44]. The rate of hearing device use among older adults with cognitive decline is also low. Cognitive decline cannot be cured, but may be delayed by controlling the modifiable risk factors such as hearing loss. Therefore we highlight the importance of hearing evaluation and rehabilitation as part of cognitive evaluation in this vulnerable population. Similarly, we suggest using the RBANS-H during the pre-implantation evaluation of CI candidates.

Finally, the WHO recommends [2] modifying lifestyle-related risk factors to lower the risk of dementia, so drawing attention to hearing loss may help globally raise awareness of these factors.

## Conclusions

Using the RBANS-H, a cognitive test adapted for people with hearing loss, we demonstrated a significant improvement in cognitive abilities in two age groups of CI users (< 60y and ≥ 60y) 12 months after cochlear implantation.

Speech perception and quality of life improved after cochlear implantation, the results being similar in both age groups. Depression scores significantly decreased in both age groups.

Age was strongly associated with the post-implantation cognitive outcomes. In the ≥ 60y subjects, the improvement in quality of life was positively correlated with cognition.

Further long-term studies are imperative to understand the long-term effects of cochlear implantation and other possible variables on cognition.
